# Configuration Design and Workspace Analysis of a Large-Scale Motion Simulator Based on Cable-Driven Parallel Technology

**DOI:** 10.3390/s26175550

**Published:** 2026-09-01

**Authors:** Fei Guo, Wanhong Lin, Jiangang Chao, Hua Deng

**Affiliations:** National Key Laboratory of Human Factors Engineering, China Astronaut Research and Training Center, Beijing 100094, China; acclwh@163.com (W.L.); xjtucjg@163.com (J.C.); dehxya@163.com (H.D.)

**Keywords:** cable-driven parallel mechanism, workspace analysis, motion simulator

## Abstract

Concentrating on a large-scale motion simulator, this article proposes the cable-driven parallel mechanism (CDPM), whose virtues are simplified geometry, being lightweight, and having a large workspace, high workload, and fast dynamic performance. To meet engineering requirements for motion simulation, we devise several CDPM configurations and establish a systematic selection procedure. The target configuration is determined based on several constraints, including the simulator’s installation space, the end-effector’s shape and load, and the required degrees of freedom (DOFs) and motion range. We calculate the wrench-closure workspace (WCW) and wrench-feasible workspace (WFW), incorporating the range of posture variations into their volume calculations. These workspaces serve as key criteria for configuration selection. Based on the WFW, we optimize the distal attachment points of the cables connected to the end-effector and adjust the minimum and maximum cable tension constraints. This optimization provides the basis for selecting the cable and motor models and ultimately establishes the optimal configuration. The proposed method encompasses the entire process, from defining large-scale motion requirements to determining the final parameters of the simulator.

## 1. Introduction

Motion simulators typically employ parallel 6-DOF kinematics, such as the Stewart platform, to reproduce flight-related sensorimotor cues. In order to obtain more realistic motion perception, the simulator requires higher acceleration, greater velocity, and a longer stroke. Among various mechanism types, the cable-driven parallel mechanism offers distinct benefits, including a larger workspace and higher end-effector speed and acceleration [1]. Compared with traditional series manipulators with rigid links, the CDPM features a simpler structure, lighter weight [2], larger workspace, lower power consumption [3], and higher stiffness.

This work focuses on the simulation of flight motion and proposes a design scheme for a large-span cable-driven parallel mechanism. The end-effector accomplishes the motion simulation tasks by actuating multiple cables. Extensive research on CDPMs has been conducted both domestically and internationally, with applications spanning crane engineering [4], astronomical observation [5], industrial manufacturing [6], aircraft painting and maintenance [7], flight simulators [8] and other fields.

The workspace of a mechanism is defined as the set of all reachable spatial points [9] and serves as a key metric for evaluating the mechanism’s performance [10]. Different mechanisms exhibit distinct workspace shapes, which depend on the interplay among the number of cables, the end-effector’s DOFs, the arrangement of cable attachment points, and the cable tension constraints [11]. To select a suitable CDPM configuration, one must compare the workspaces of candidate configurations [12] and verify whether their translational and rotational ranges meet the flight simulation requirements.

Cables can only transmit tension, not compression, due to their unidirectional force property [13]. To account for this unidirectional property and the associated tension limits, researchers [14] have defined several workspace types, including the static-equilibrium workspace (SEW), wrench-closure workspace (WCW) [15], force-feasible workspace (FFW) [16], dynamic workspace (DW), and collision-free workspace (CFW). Pusey [17] defined the SEW as the statically reachable workspace; however, not all end-effector configurations are statically reachable. Roberts [18] calculated the SEW for a CDPM in a specific configuration using the static-equilibrium equation and the null space of the Jacobian matrix. Ida [19] introduced a novel algorithm for computing the SEW of underactuated CDPRs considering tension distribution. Verhoeven [20] proposed the definition and analytical expression of the CFW using a semi-algebraic set. For an incompletely restrained positioning mechanism, researchers employed the vector geometry method, treating the end-effector as an equivalent mass point [21]. This method yielded the analytical formulation of the workspace but could not characterize the workspace with respect to the end-effector’s posture angles. For a redundantly restrained positioning mechanism, the cable tension equilibrium equation is transformed into several subproblems using an elimination method [22], in which the pose points are evaluated by checking the signs of the resulting expressions to determine whether they lie within the workspace. Combining the convex theory with the Jacobian matrix, Diao [23] solved the WCW of the CDPM using a numerical approach. Gouttefarde [24] calculated the WFW of the CDPM using an interval analysis method. Xin [25] developed a calculation method for the WCW based on workspace existence conditions and a unified solution strategy. Gosselin [26] proposed the concept of the DW and developed a calculation routine that incorporates acceleration to broaden the motion range of the end-effector. Ferraresi [27] introduced a specialized calculation method for a CDPM with 6 DOFs actuated by nine cables. However, this method is not generalizable to other types of CDPM configurations.

Numerical research on workspaces has yielded encouraging results in terms of size, shape, and cable–environment collision avoidance, albeit at the cost of a large number of actuators. Williams [28] proposed a concept for a CDPM actuated by 12 cables with linearly movable proximal anchor points. Korayem [29] investigated the workspace of a suspended CDPM with elastic and heavy cables, thereby accounting for the effect of cable sagging. Most prior studies have focused on generic algorithms or small-scale robots. However, a systematic design workflow for large-scale motion simulators—one that combines configuration selection, WCW/WFW analysis, and parametric optimization—has not yet been reported.

For the purpose of flight simulation, this work focuses on CDPM technology. The proposed CDPM is intended for motion cueing in exercise-based flight simulators, where rapid, large-stroke motions are essential for vestibular perception. Its cable-based actuation not only reduces moving inertia and power consumption but also substantially lowers procurement and maintenance costs compared with conventional hydraulic platforms. The first stage involves designing an appropriate mechanism tailored to the flight motion simulator’s requirements. This stage includes calculating the wrench-closure workspace (WCW) and wrench-feasible workspace (WFW) for six configurations, which differ in the number and arrangement of cables. In addition to the hardware composition and mechanism design of the motion simulator, the kinematics and force equilibrium are also addressed. The effects of different postures, distal attachment point locations (cables connected to the end-effector), and cable tension constraints on the CDPM are demonstrated. By tuning these influencing parameters, an optimal configuration of the CDPM simulator can be obtained.

## 2. Motion Simulator Design

**Figure 1:**
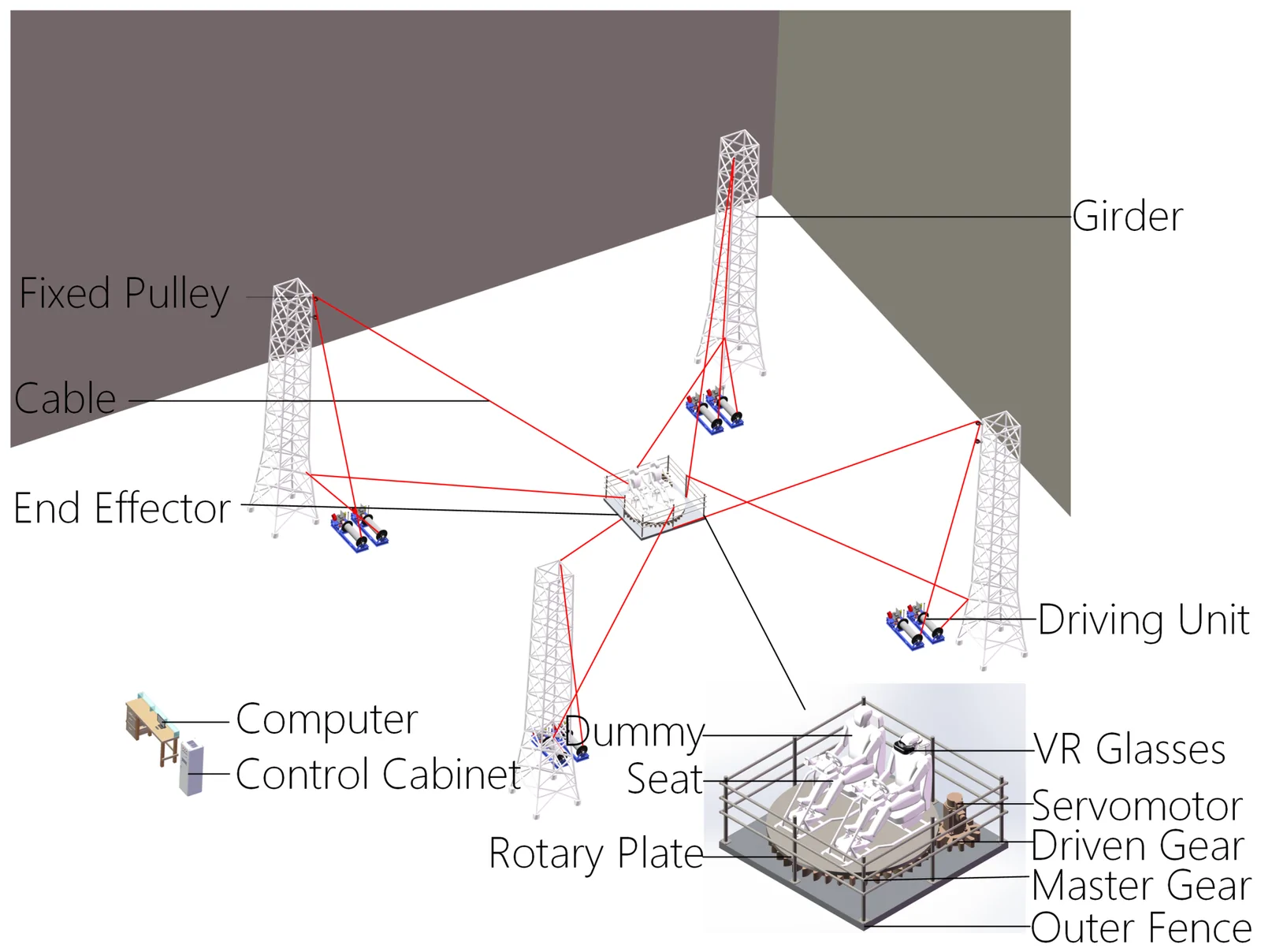
Framework of motion simulator based on cable-driven parallel mechanism.

As illustrated in Figure 1, the motion simulator is composed of a basic frame, a cable-driven system, and an end-effector. The basic frame includes a truss, fixed pulleys, and cables. The cable-driven system consists of a servo motor, a reduction gear, a winding drum, a control cabinet, and a computer. A dummy with VR glasses fixed on it is mounted on the end-effector to conduct visual simulation. The end-effector, designed with a larger rotational range, comprises a single-degree-of-freedom servomotor, a driven gear, a driving gear, and a rotary plate.

The geometric parameters and motion requirements of the cable-driven parallel simulator are summarized in Table 1. The angular ranges are derived from representative flight simulation scenarios. The feasible cable tension range is initially estimated based on actuator capabilities and cable strength and will be further optimized in Section 5.1 to balance workspace size against hardware cost.

For a CDPM, achieving full restraint with *n* degrees of freedom requires at least n+1 cables [30]. Based on the relationship between the number of cables *m* and the degrees of freedom of the end-effector *n*, CDPMs can be classified into three types, namely, incompletely restrained positioning mechanisms (m<n+1, IRPMs), completely restrained positioning mechanisms (m=n+1, CRPMs), and redundantly restrained positioning mechanisms (m>n+1, RRPM) [31].

**Figure 2:**
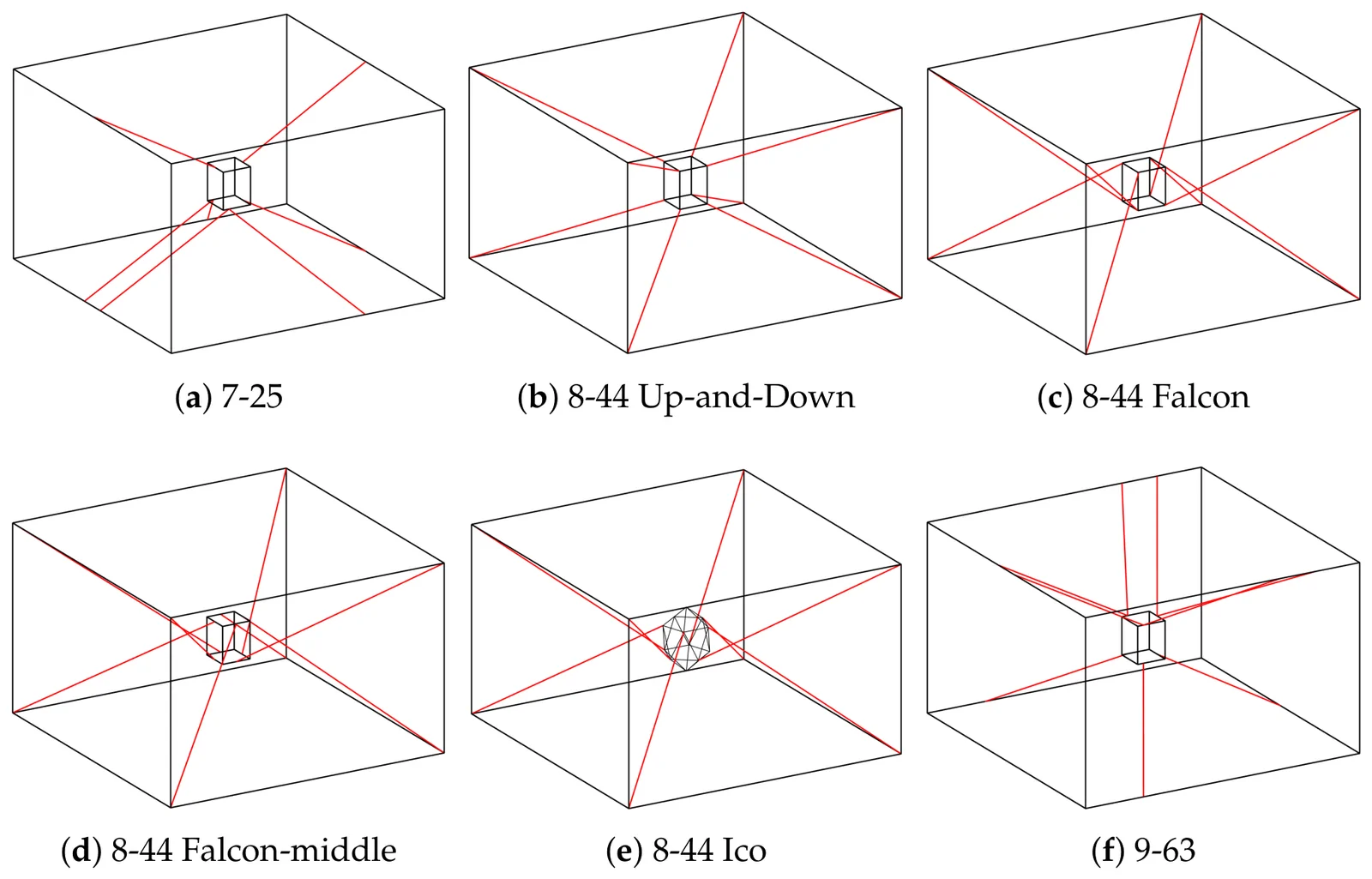
Different configurations of CDPM.

The design responds to the demands of a large-scale motion simulator, including the installation space defined by proximal anchor points, the end-effector geometry, load capacity, motion DOFs, and the desired posture range. Since the motion simulator requires 6 DOFs (as listed in Table 1), the configuration must be either a CRPM or an RRPM. When external forces act on the end-effector, a redundantly restrained driving method can be adopted. However, the redundantly restrained configuration introduces several challenges in mechanical analysis and control strategy, including increased risks of cable-to-cable and cable-to-end-effector interference. As the number of cables increases, the force distribution algorithm for redundantly restrained systems becomes more complex. Therefore, considering cable tension and workspace constraints, we select the target mechanism from CDPM configurations with 7 to 9 cables and subsequently perform further optimization. Figure 2 illustrates six CDPM configurations: 7-25 (two upper and five lower cables), 8-44 Up-and-Down, 8-44 Falcon (four upper and four lower cables arranged in a staggered pattern), 8-44 Falcon-middle (where the distal attachment points are not located at the vertices of the end-effector), 8-44 Ico (with an icosahedral end-effector), and 9-63. In the following section, we take these configurations as examples to analyze their workspaces and optimize their structures.

## 3. Kinematic Model and Static-Equilibrium Analysis

**Figure 3:**
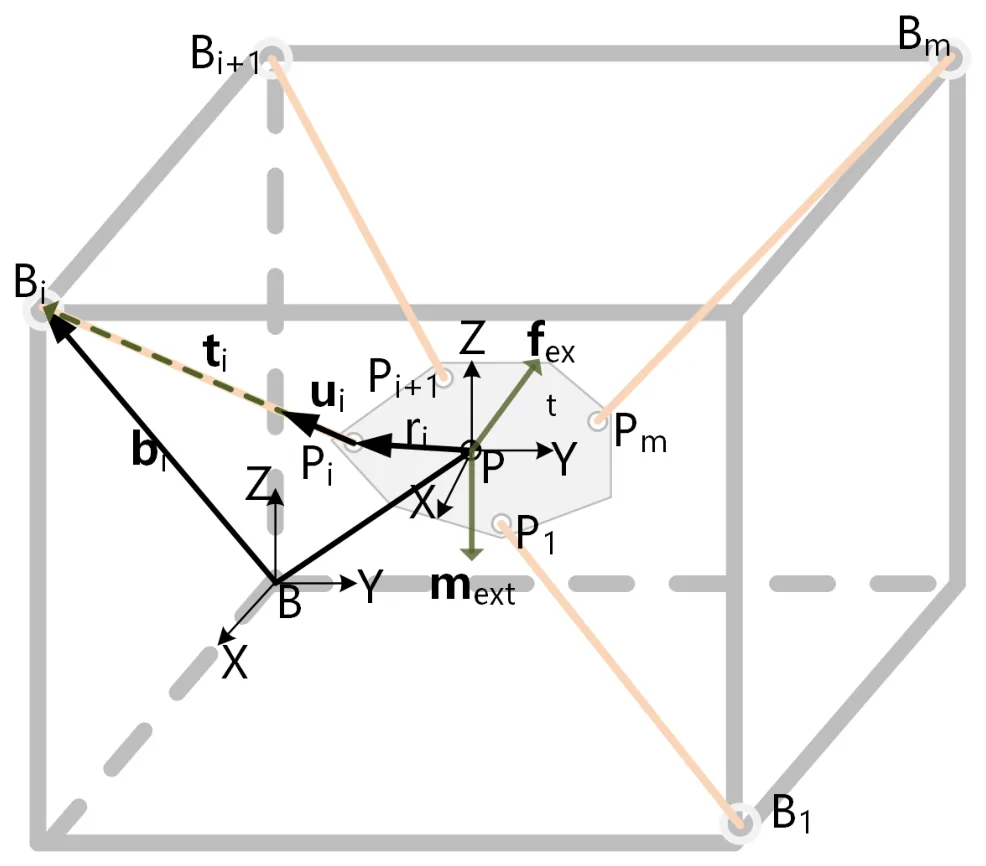
Kinematic and kinetostatic description of CDPR actuation by m cables.

Figure 3 illustrates the kinematic and kinetostatic description of a cable-driven parallel mechanism with n degrees of freedom (DOFs) actuated by *m* cables.

### 3.1. Kinematic Model

To analyze the kinematic model [9], we construct a simplified model with two coordinate frames: a fixed frame $K_{B}-OXYZ$ attached to the base, and a moving frame $K_{P}-PXYZ$ attached to the end-effector. Here, $B_{i}$ denotes the proximal anchor point, and $P_{i}$ denotes the corresponding distal attachment point on the end-effector. The cable length $l_{i}$, defined as the distance between $B_{i}$ and $P_{i}$, determines the pose X(x,y,z,θ,ϕ,ψ) of the moving end-effector relative to the base frame. The vector $r_{i}$ denotes the vector from the end-effector center P to $P_{i}$. The unit vector along the cable can then be calculated from the following equation:

(1) $$u_{i}=\frac{L_{i}}{l_{i}}$$

Based on the simplified model of the cable-driven parallel mechanism illustrated in Figure 3, the cable lengths can be calculated using the vector closure principle.

(2) Li=OBi−OP−RPO·PPi

where OP denotes the position vector of the end-effector center in the fixed base frame. ${\mathrm{OB}}_{i}$ indicates the position vector of proximal anchor points in the fixed base frame. ${\mathrm{PP}}_{i}$ signifies the position vector of the distal attachment points in the moving end-effector frame. RPO denotes the rotation matrix from the moving end-effector frame to the fixed base frame.

(3) RPO=cϕcψ−cϕsψsϕsθsϕcψ+cθsψ−sθsϕsψ+cθcψ−sθcϕ−cθsϕcψ+sθsψcθsϕsψ+sθcψcθcϕ

in which s=sin and c=cos.

The length of the $i_{th}$ cable can be calculated through

(4) $$l_{i}=\sqrt{L_{i}^{T}L_{i}}$$

Therefore, the inverse kinematic equation is derived to compute each cable length $l_{i}$ given the known end-effector configuration X.

### 3.2. Kinetostatic Analysis

Compared with the end-effector mass and the large cable tensions, the cable masses and frictional effects can be considered negligible. For the kinetostatic condition [18] of the end-effector, the force equilibrium equation can be formulated as

(5) $$\sum_{i=1}^{m}u_{i}\cdot t_{i}+f_{ext}=0.$$

(6) $$\sum_{i=1}^{m}r_{i}\times \left(u_{i}\cdot t_{i}\right)+m_{ext}=0$$

According to the two formulations derived above, we obtain the following:

(7) $$\left[\begin{array}{cccc} u_{1} & u_{2} & \cdots & u_{m}\\ r_{1}\times u_{1} & r_{2}\times u_{2} & \cdots & r_{m}\times u_{m} \end{array}\right]T+\left[\begin{array}{c} f_{ext}\\ m_{ext} \end{array}\right]=0$$

That is,

(8) JT+W=0

in which $T=[t_{1};t_{2};\ldots ;t_{m}]$ signifies an m×1 tension vector in the cables, and J is the structure matrix, which depends on the end-effector configuration X.

The cable tension constraint should satisfy the force equilibrium condition of the cable’s unidirectional loading requirement. The value of cable tension is between the maximum force that the cable can withstand and the minimum force that can be applied with the cable remaining taut, which are the ceiling and floor criteria related to cable material characteristics.

### 3.3. Workspace Generation Parameters

#### 3.3.1. Coordinates of Proximal Anchor Points on Base Frame

For the 20 m × 20 m × 11 m frame, the proximal anchor points ($B_{1}$ to $B_{8}$) are provided in the following. For example, for the 8-44 configurations:

- The coordinates of the top points in the base frame: $B_{1}=\left(-10,-10,11\right)$, $B_{2}=\left(10,-10,11\right)$, $B_{3}=\left(10,10,11\right)$, $B_{4}=\left(-10,10,11\right)$;
- The coordinates of the bottom points in the base frame: $B_{5}=\left(-10,-10,0\right)$, $B_{6}=\left(10,-10,0\right)$, $B_{7}=\left(10,10,0\right)$, $B_{8}=\left(-10,10,0\right)$.

#### 3.3.2. Coordinates of Distal Attachment Points on End-Effector

For the 2 m × 2 m end-effector, the distal attachment points ($P_{1}$ to $P_{8}$) are provided. For the Falcon and square end-effectors, attachment points are different:

- Icosahedron: $P_{1}=\left(-1.23,-0.895,4.75\right)$, $P_{2}=\left(0.47,-1.45,4.75\right)$, $P_{3}=\left(1.52,0,4.75\right)$, $P_{4}=\left(-1.23,0.90,4.75\right)$ (bottom frame); $P_{5}=\left(-0.47,1.45,6.3\right)$, $P_{6}=\left(1.23,-0.9,6.3\right)$, $P_{7}=\left(-0.47,1.45,6.3\right)$, $P_{8}=\left(1.23,0.9,6.3\right)$ (top frame);
- Square: $P_{1}=\left(-2,-2,4.5\right)$, $P_{2}=\left(2,-2,4.5\right)$, $P_{3}=\left(2,2,4.5\right)$, $P_{4}=\left(-2,2,4.5\right)$ (top frame); $P_{1}=\left(-2,-2,6.5\right)$, $P_{2}=\left(2,-2,6.5\right)$, $P_{3}=\left(2,2,6.5\right)$, $P_{4}=\left(-2,2,6.5\right)$ (bottom frame).

#### 3.3.3. End-Effector Geometry and Inertial Parameters

- Platform geometry: Square platform with edge length 2 m;
- Mass: Maximum payload 1000 kg (including dummy and VR equipment);
- Inertia tensor: $I_{xx}=I_{yy}=I_{zz}=200$ kg·m^2^ (estimated based on payload distribution).

#### 3.3.4. Euler-Angle Convention

We use the Z-Y-X Euler-angle convention (intrinsic rotations about *Z*, *Y*, and *X* axes) for representing end-effector orientation. The rotation matrix is defined as $R=R_{z}\left(\phi \right){\dot{R}}_{y}\left(\theta \right){\dot{R}}_{x}\left(\psi \right)$, where ϕ, θ and ψ correspond to roll, pitch, and yaw angles, respectively.

#### 3.3.5. External Wrench Definition

The external wrench $W={\left[f_{x},f_{y},f_{z},\tau_{x},\tau_{y},\tau_{z}\right]}^{T}$ includes

- Gravity: $f_{z}=-mg=-9800$ N (at center of mass);
- Other external forces and moments: Assumed to be zero for static analysis (the simulator is not subjected to aerodynamic or contact forces during motion).

## 4. Workspace Analysis

In this paper, we adopt the standard terminology established in the CDPM literature [14,16,20,23,24]. The wrench-closure workspace (WCW) is defined as the set of end-effector poses for which positive cable tensions can balance any external wrench, whereas the wrench-feasible workspace (WFW) denotes the set of poses for which cable tensions can be maintained within prescribed lower and upper bounds.

### 4.1. Wrench-Closure Workspace Analysis and Calculation

The wrench-closure workspace (WCW) of a cable-driven parallel mechanism is defined as the set of end-effector configurations for which there exists a set of positive cable tensions that can balance any arbitrary wrench applied to the end-effector. Following the standard definition in the literature, WCW analysis considers only gravity as the external wrench. This definition yields a conservative workspace estimate: any pose within the WCW can theoretically balance any external wrench—including inertial loads—as long as the cable tensions can be adjusted within positive bounds. The WCW can be expressed mathematically as follows:

(9) $$\forall W\in R^{n}\;,\exists T\geq 0:JT+W=0$$

in which W is an n×m matrix.

To attain the above wrench-closure condition, a necessary condition is Rank[J] = n, with m≥n+1. The following equivalent statements hold:

- The configuration is wrench-closure.
- The column vectors of J positively span a convex hull that contains a neighborhood of the origin.
- There does not exist a nonzero vector $a\in R^{1\times n}$, a≠0, such that $a\cdot j_{i}\geq 0$ for i=1,…,m.
- The column vectors of the structure matrix J positively span $R^{n}$.

**Figure 4:**
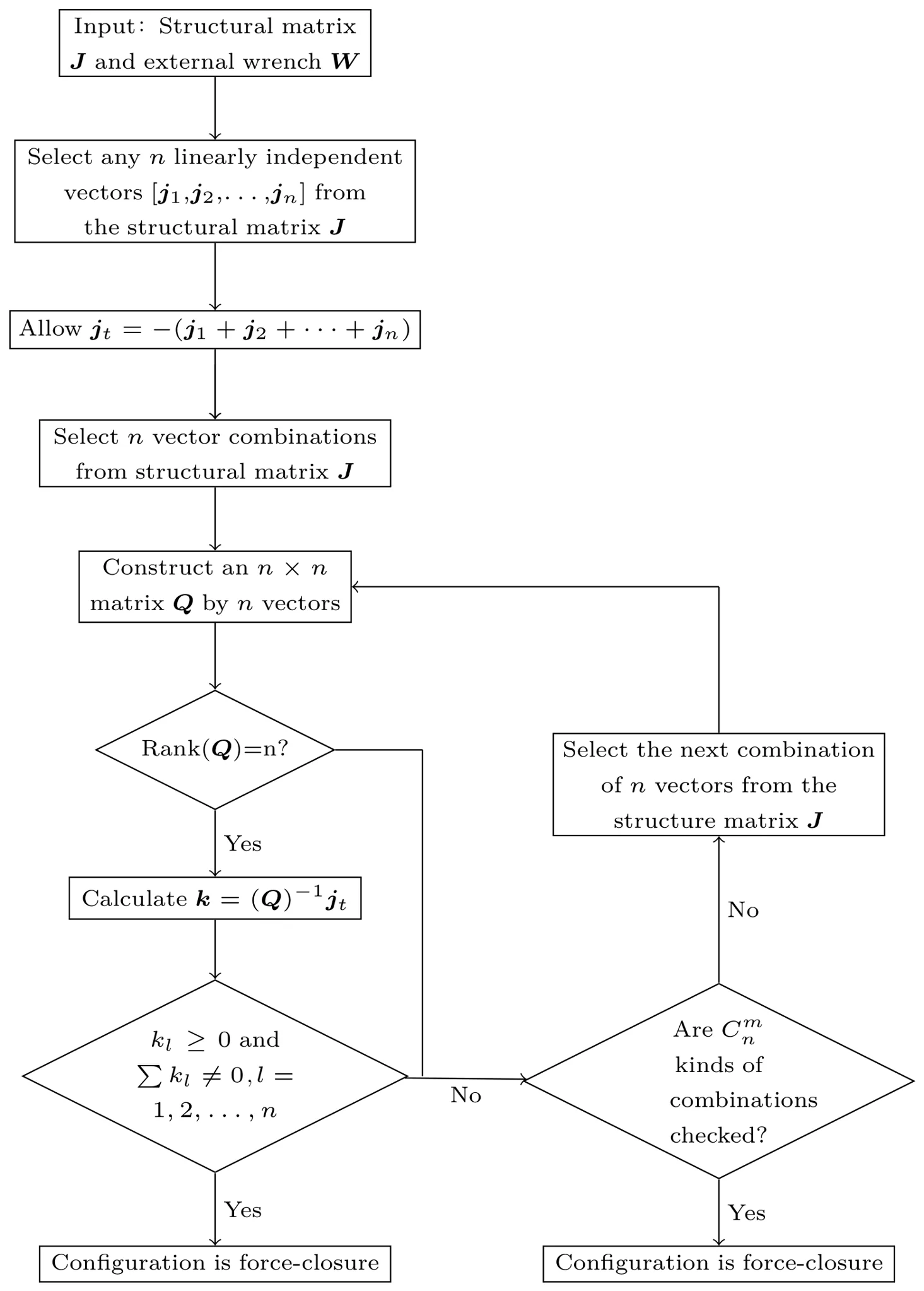
Flow chart of WCW analysis algorithm.

For an n×m structure matrix J with rank *n*, the wrench-closure configuration is attained if and only if there exists a vector $j_{t}=-\left(j_{1}+j_{2}+\cdots +j_{n}\right)$ that can be positively represented by at least one set of *n* linearly independent column vectors chosen from $j_{i}\left(i=1,2,\cdots ,m\right)$. The procedure for calculating the wrench-closure workspace of a completely restrained CDPR is illustrated in Figure 4 and consists of the following steps:

1. Select a set of *n* linearly independent vectors $j_{1},j_{2},\cdots ,j_{n}$ from the column vectors of J.
2. Form the vector $j_{t}=-\left(j_{1}+j_{2}+\cdots +j_{n}\right)$ from the selected set.
3. Form a combination of *n* vectors from the column vectors of J to construct an n×n matrix Q, whose columns are the *n* selected vectors.
4. Verify that rank[Q]=n; otherwise, terminate the procedure, as the configuration is not wrench-closure.
5. Obtain a vector k through $Q\left[\begin{array}{c} k_{1}\\ k_{2}\\ \ddots \\ k_{n} \end{array}\right]=j_{t}$; therefore $\left[\begin{array}{c} k_{1}\\ k_{2}\\ \ddots \\ k_{n} \end{array}\right]=Q^{-1}j_{t}$.
6. If $k_{j}\geq 0$ and $\sum k_{j}\neq 0$ for all j=1,2,⋯,n, then the configuration satisfies the wrench-closure condition. If this is the final configuration to check, stop; otherwise, proceed to Step 7.
7. Repeat the process starting from Step 3 until all $C_{n}^{m}$ combinations have been checked. If none of the combinations satisfies the condition in Step 6, then the end-effector configuration is not wrench-closure.

**Figure 5:**
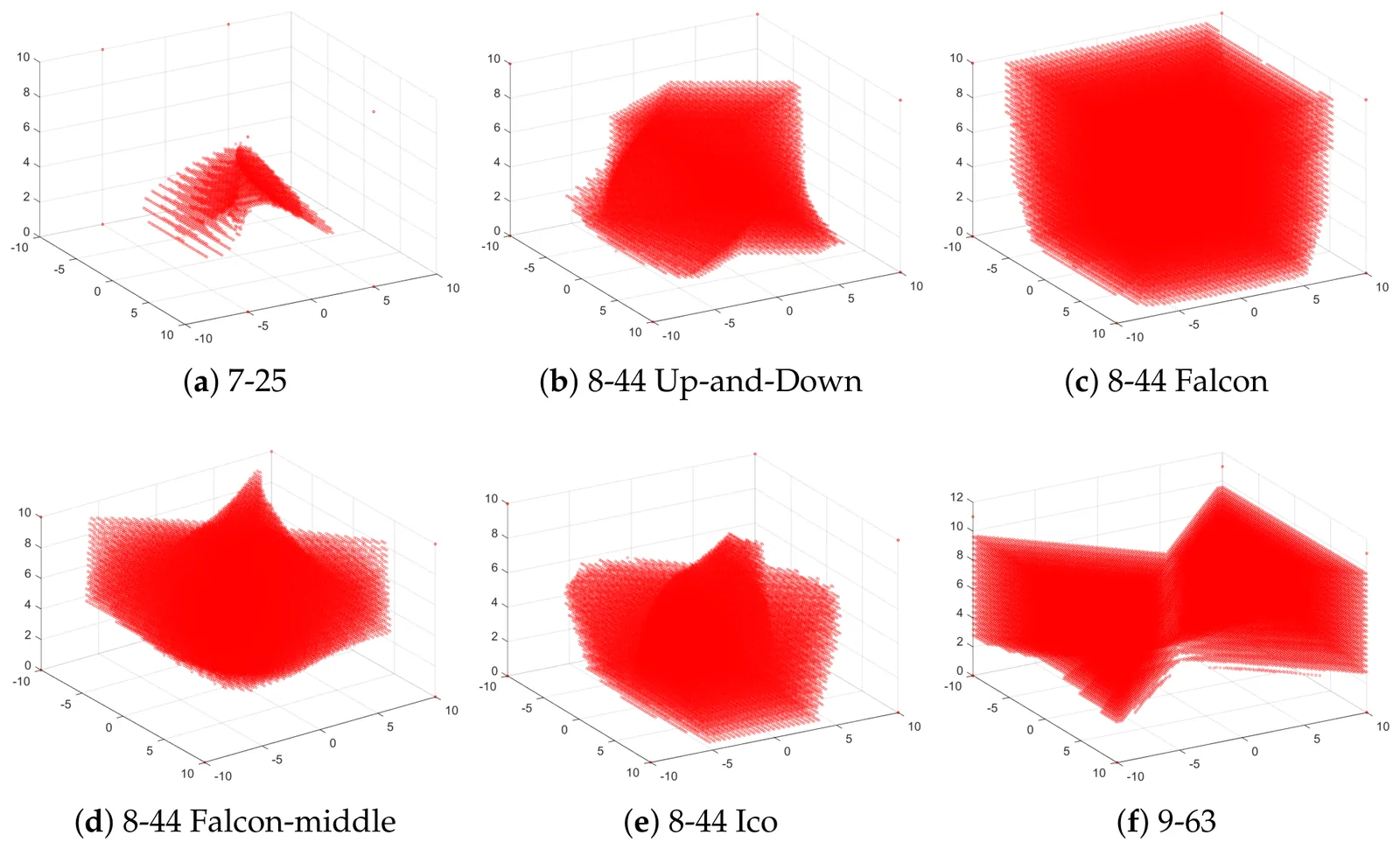
WCW of CDPM with different configurations.

Using the above algorithm, we traversed all points within the frames defined by the cable anchor points for different configurations. The displacement and posture sampling intervals were 0.2 m and 2°, respectively. For each sampled point, we determined whether it is wrench-closure. The number of WCW points for each configuration is listed in Table 2, and the corresponding workspaces are visualized in Figure 5.

As can be seen in Table 2, the number of WCW points for the six configurations, in descending order, ranks as follows: (c), (f), (d), (e), (b), and (a). Generally, the number of sample points serves as an indicator of the WCW size. Figure 5 presents the WCW visualizations for the different configurations. Although configuration (f) ranks second in point count, the middle portion of its workspace is relatively narrow, which may introduce several singularities. Configurations (c), (d), and (e) exhibit WCWs that are narrow at the top and bottom but wide in the middle. Consequently, the system exhibits high load capacity when the end-effector is near the central position. For the eight-cable configurations (b–e), the lower portion of the WCW is smaller than the upper portion. During operation, the end-effector of the simulator is subjected only to gravity and cable tensions. In the WCW calculation, the external force $f_{ext}$ includes only gravity, which is consistent with both theoretical definitions and practical requirements. It can be inferred that the system exhibits a higher load capacity when the end-effector is above the central position than when it is in the lower zone. This is because the downward cable force has a larger vertical component to counteract the external force $f_{ext}$. When the end-effector moves to the lower zone, the vertical component of the downward cable force becomes smaller. Consequently, the load capacity against the external force $f_{ext}$ decreases accordingly. A comparison between configurations (b) and (c) reveals that the cross-connection between the upper and lower cables can enlarge the workspace and is beneficial for balancing a larger external $f_{ext}$. Hence, the cross-connection configuration can expand the effective workspace.

The point clouds shown represent the results of an offline workspace sampling; no dynamic simulation was involved. The computation took approximately 5 s per configuration, with a computational complexity of $O(N_{d}*m^{3})$ for the workspace, where *N* is the number of sampling points, *d* is the degrees of motion freedom, and *m* is the number of cables. The actual computation time depends on the computer’s hardware performance.

### 4.2. Wrench-Feasible Workspace Analysis and Calculation

According to Equation (9), the wrench-closure workspace (WCW) does not account for tension limits on the cables. In practice, the dynamic torques from actuators and the maximum allowable cable forces define an upper safety limit. Additionally, to prevent cables from slacking and to maintain a certain mechanism stiffness, the cable forces must be kept above a minimum tension threshold. Thus, the cable tension constraints can be expressed as

(10) $$0<T_{min}\leq T\leq T_{max}\left(i=1,2,\cdots ,8\right)$$

Based on the WCW, the wrench-feasible workspace (WFW) is defined as the set of end-effector poses for which there exists a set of cable tensions satisfying both the equilibrium equation and the tension bounds.

(11) $$\forall W\in R^{n}\;,\exists T\in \left[T_{min},T_{max}\right],T_{min}\geq 0:JT+W=0$$

The force distribution calculation for the WFW can be formulated by decomposing the cable tension vector T into two components:

(12) $$T=T_{m}+T_{v}$$

where $T_{v}$ represents the particular solution, and $T_{m}=\frac{T_{min}+T_{max}}{2}$ expresses the mean tension vector. Substituting Equation (12) into Equation (8) yields the transposed structure matrix $J^{T}\in R^{m\times n}$ and the following equation:

(13) $$J^{T}T_{v}=-W-J^{T}T_{m}$$

Multiplying both sides of the above equation by $J^{T}$ and $J^{T+}$, respectively, yields

(14) $$T=T_{m}-J^{T+}\left(W+J^{T}T_{m}\right)$$

**Figure 6:**
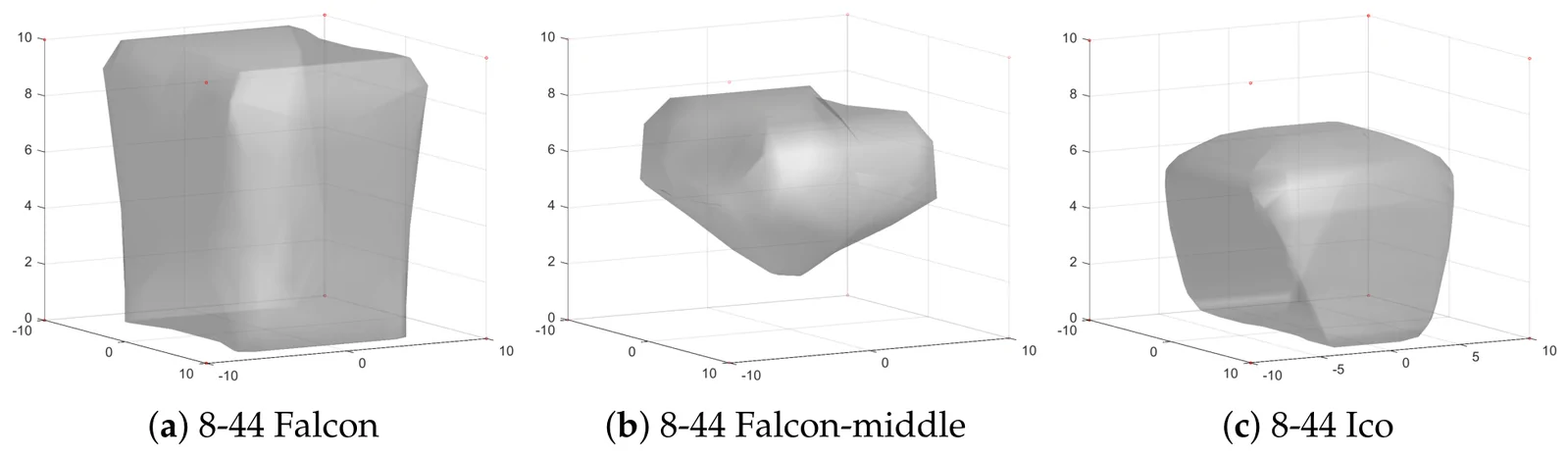
WFW of CDPM with different configurations.

The minimum and maximum cable tensions are set to 1000 N and 28,000 N, respectively. The WFW hulls for the six configurations are then computed, which include all points satisfying Equations (11) and (14). As is shown in Figure 6, the WFW excludes several points compared with the WCW. The WFW is a subset of the WCW.

### 4.3. The WFW of Different Configurations with Posture Variations

**Figure 7:**
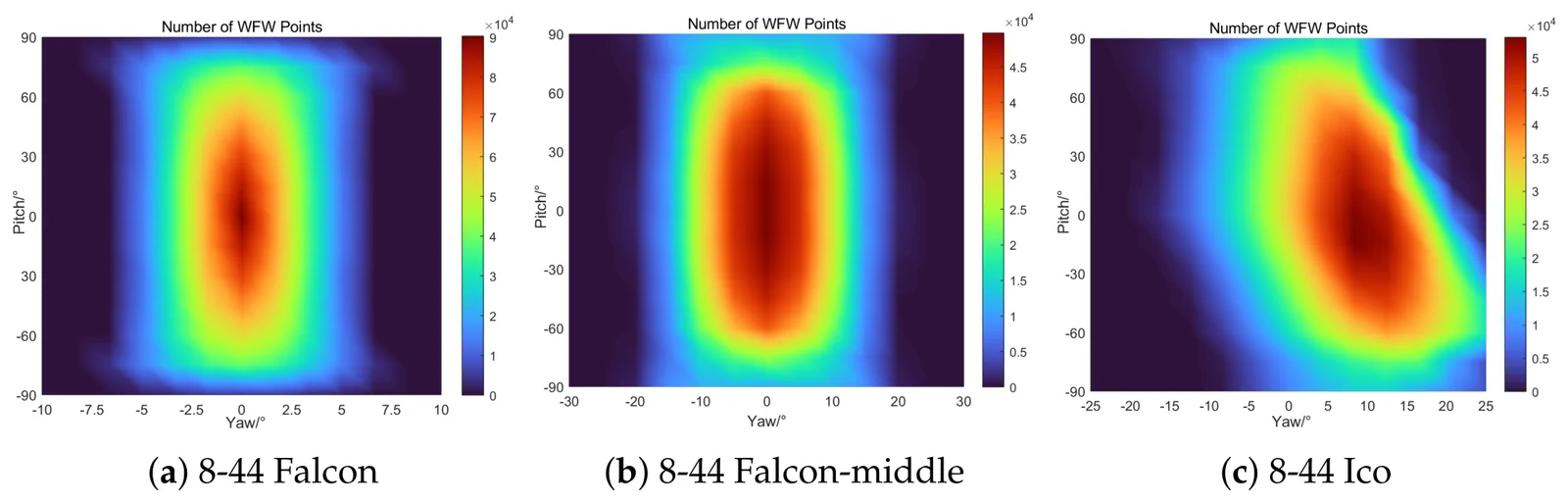
WFW point distributions under posture variations for different CDPM configurations.

The above-mentioned WFW is valid only when the posture angles of the end-effector are 0. To investigate how posture variations affect the WFW of different configurations, we conducted the following analysis. The WFW distributions for three selected configurations under varying postures are visualized using thermal maps in Figure 7. Configurations (a) and (c), which have the largest workspaces, are selected as candidates for further evaluation. In our flight simulation task, the roll angle must be controlled and maintained at 0° to ensure balance. For the three selected configurations, the pitch and yaw angle ranges are as follows: pitch ∈[−75°,75°] and yaw ∈[−5°,5°]; pitch ∈[−70°,70°] and yaw ∈[−15°,15°]; and pitch ∈[−70°,80°] and yaw ∈[−10°,22°], respectively. All three configurations satisfy the required posture ranges. The 8-44 Falcon configuration (configuration (a) in Figure 7) exhibits the largest WFW and is therefore selected as the end-effector for the motion simulator.

## 5. Influence Factor Analysis of Wrench-Feasible Workspace

### 5.1. The Cable Tension Constraint

**Figure 8:**
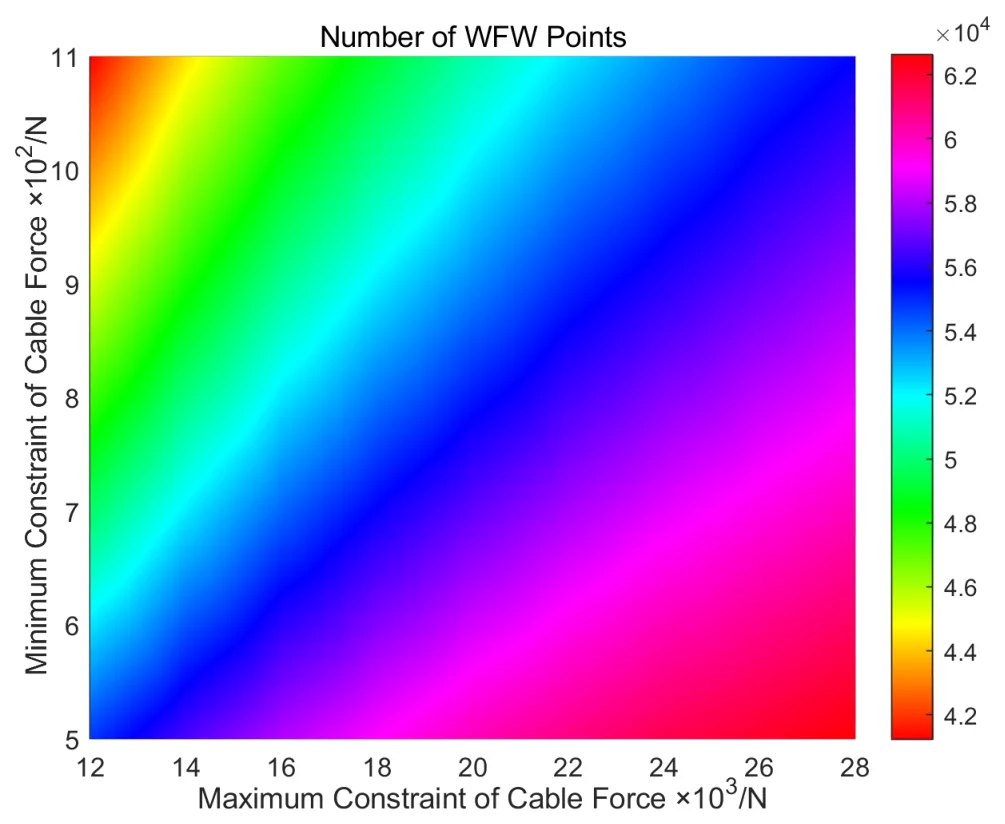
WFW point distribution under varying minimum/maximum cable tension constraints for the 8-44 Falcon configuration.

Equation (10) defines the cable tension constraints. As the allowable cable tension range varies, the WFW changes accordingly. By adjusting the minimum and maximum cable tension constraints, we evaluate the influence of the tension range on the WFW, as shown in Figure 8. A lower minimum tension bound and a higher maximum tension bound both enlarge the WFW. Therefore, widening the allowable cable tension range can effectively expand the WFW. Meanwhile, the upper tension limit is constrained by the actuator driving torque and the cable’s allowable tensile strength. A larger actuating force from the driving motor and higher system rigidity tend to produce a larger WFW. However, this generally leads to higher manufacturing costs. In addition, a practical lower tension bound must be maintained to keep the cables taut and to ensure sufficient system stiffness. Based on the above considerations, the cable tension constraints are set to [1000 N, 24,000 N]. Consequently, selecting appropriate hardware specifications—including motors and cables—to achieve a suitable cable tension range can effectively improve the mechanism’s performance while minimizing cost.

When selecting motor and cable specifications, we apply a safety factor of 1.5 to ensure that the theoretical calculations remain valid in practical engineering applications. For example, the maximum torque that the driving unit must provide becomes 24,000 N × 1.5 = 36,000 N.

Using a safety factor of 1.5 is a common engineering practice in the design of cable-driven mechanisms and motion simulators, and it is chosen based on the following considerations:

A. Dynamic load amplification: During dynamic operation, particularly under acceleration and deceleration, the actual cable tensions may exceed static values. A factor of 1.5 accounts for typical dynamic load amplifications in moderate-speed motion simulators.
B. Cable fatigue and wear: Cables experience cyclic loading during repeated operations, and a safety margin helps extend their service life by keeping operating stresses well below the ultimate tensile strength.
C. Uncertainty in friction and manufacturing tolerances: Pulley friction, cable bending losses, and manufacturing tolerances introduce additional uncertainties. A safety factor of 1.5 provides a buffer against these unmodeled effects.
D. Industry standards: In the design of cable-driven parallel robots and motion platforms, safety factors between 1.5 and 2.0 are commonly adopted. For example, the IPAnema system family uses similar safety margins for industrial applications. Our choice of 1.5 represents a conservative yet economically reasonable value that balances safety and cost.

### 5.2. Effect of Distal Attachment Point Location

**Figure 9:**
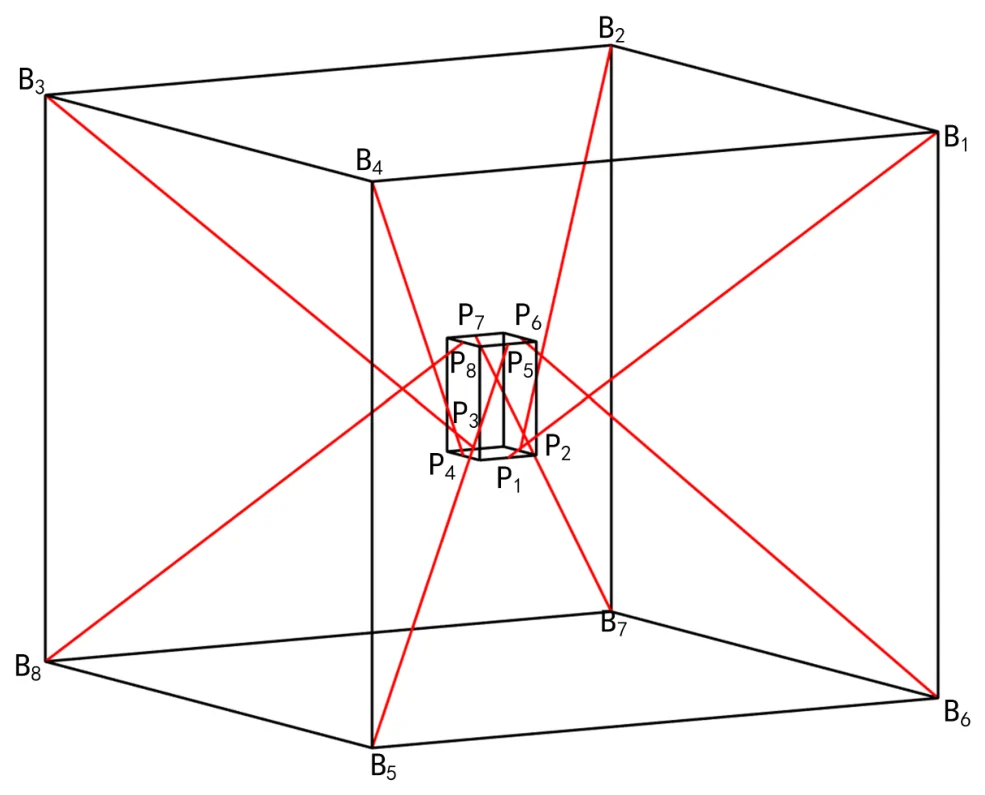
Cable geometry of 8-44 Falcon configuration.

When cable-to-cable interference occurs, the motion of the end-effector is restricted, which impedes the normal operation of the CDPM. Therefore, adjacent cables must maintain a sufficient clearance distance. As illustrated in Figure 9 (cf. Figure 2c), the distal attachment points are moved from the vertices to the edge centers of the end-effector.

**Figure 10:**
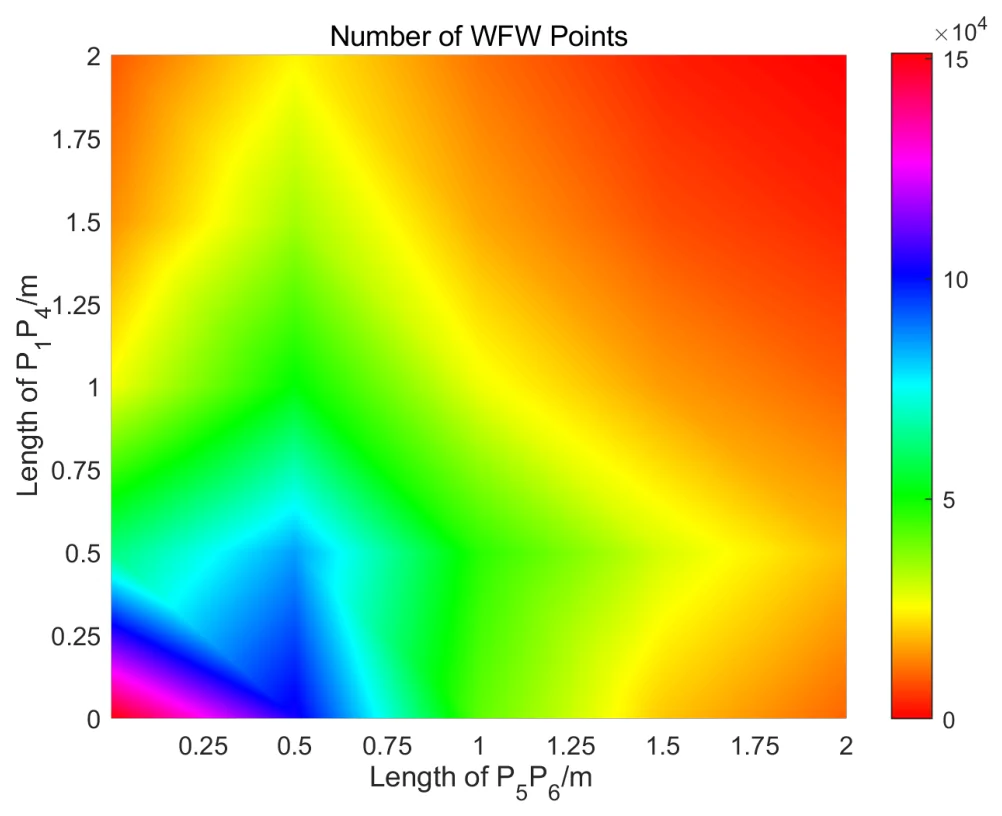
WFW point distribution for varying distal attachment point locations of the 844 Falcon configuration.

We optimize the distal attachment point locations of the cables connected to the end-effector. The distances between connector pairs ($P_{1}-P_{4}$ and $P_{2}-P_{3}$) are set to vary from 0 to 2 m. Similarly, the distances between connector pairs ($P_{5}-P_{6}$ and $P_{7}-P_{8}$) are also set to vary from 0 to 2 m. The number of WFW points for different distal attachment point locations is presented in Figure 10.

As inferred from Figure 10, as the distances between $P_{1}-P_{4}$ and $P_{5}-P_{6}$ decrease, the WFW expands. However, a smaller distance between connector pairs also leads to a reduced posture variation range. Therefore, selecting appropriate distances between connector pairs enables a CDPM configuration suitable for the flight simulator.

The elastic elongation is calculated using the following equation:

(15) $$\Delta L=\frac{F\cdot L}{A\cdot E}$$

in which *F* is the axial tension (here set to 28,000 N), *L* is the original cable length, *A* is the effective cross-sectional area, and *E* is Young’s modulus.

Therefore, for a typical steel cable with a diameter of 12 mm, a Young’s modulus of 2 $\times 10^{11}$ Pa, and a length of 10 m, the elastic elongation under a tension of 28,000 N is approximately 2.8 cm. This elongation is less than 0.3% of the 11 m frame dimension and can be effectively compensated for by closed-loop control. Therefore, cable stiffness does not compromise the validity of the selected configuration for the intended motion simulator application.

Furthermore, cable stiffness can affect the system, causing a small position offset of the end-effector when subjected to external forces. As a result, a minor deviation exists between the actual and theoretical workspace. This deviation is related to the cable stiffness, end-effector position, and cable tension, which are strongly coupled.

## 6. Simulation

To validate the theoretical workspace analysis and verify the viability of the selected 8-44 Falcon configuration for the large-scale motion simulator, we conducted numerical simulations using MATLAB (R2023b). The simulation framework integrates the kinematic model from Section 3.1, the static-equilibrium equations from Section 3.2, and the tension distribution algorithm (Equation (14)). This section presents the simulation setup, the trajectory tracking results, and the cable tension verification.

**Figure 11:**
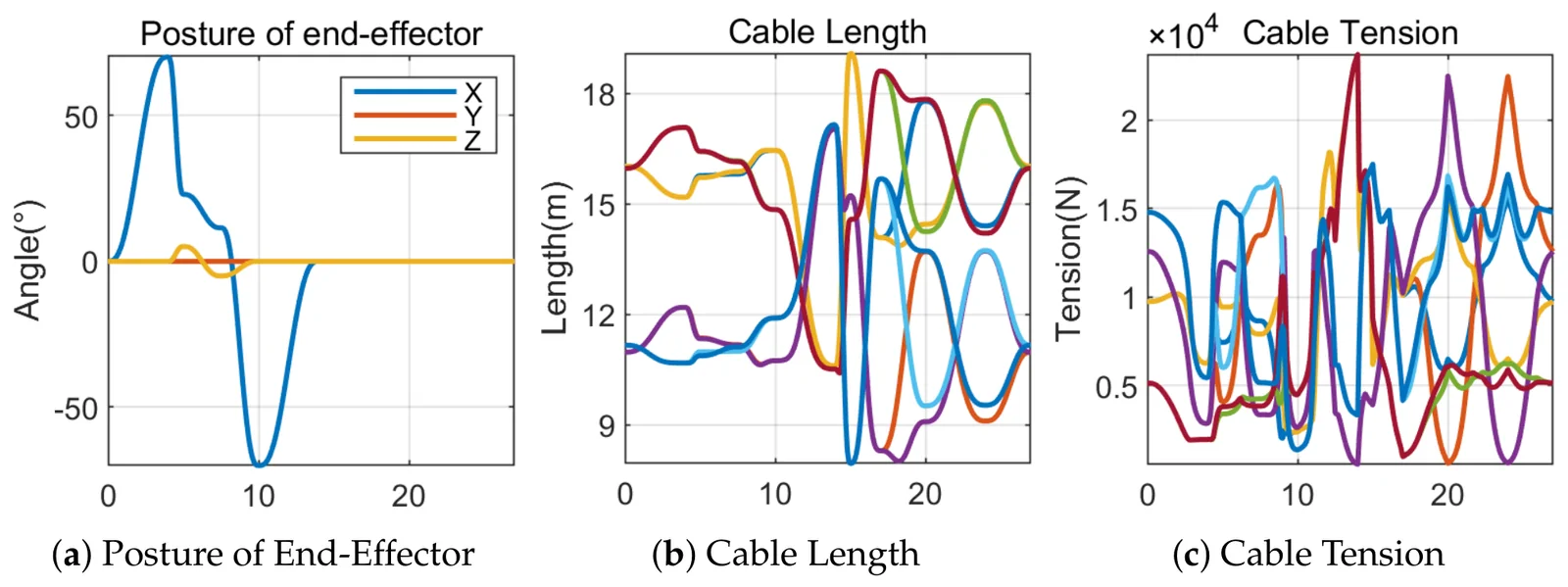
Motion simulation of end-effector for 8-44 Falcon configuration.

As shown in Figure 11a, the pitch angle varies from −68.3° to 65.6° and the yaw angle from −5.4° to 5.6°, while the roll angle is maintained at 0°, consistent with the flight workspace analysis.

To verify that the cable tensions remain within the feasible range throughout the trajectory, we computed the tension distribution for all eight cables at each simulation time step using Equation (14). Figure 11c shows the tension profiles of cables 1–8 over the 20 s simulation. All cable tensions remained strictly within the prescribed bounds [1000 N, 28,000 N], with a minimum tension of 1025 N (observed at t = 13.9 s for cable 4) and a maximum tension of 23,725 N (observed at t = 13.9 s for cable 7). Figure 11 summarizes the tension data for all eight cables, which indicate that the tension distribution algorithm effectively utilizes the available tension range without violating the constraints.

As an additional consistency check, we randomly sampled 5,000 pose points within the theoretically analyzed WFW of the 8-44 Falcon configuration (as determined in Section 4.2) and tested whether each pose satisfied the static-equilibrium condition with cable tensions within [1000 N, 28,000 N]. The results showed that all 5,000 sampled points satisfied all constraints, confirming that the theoretical WFW computation is highly reliable. This high consistency provides strong confidence that the theoretical workspace analysis accurately reflects the actual feasible workspace of the system.

## 7. Conclusions

In this paper, a large-scale motion simulator based on cable-driven parallel technology is proposed. Based on the geometric characteristics, the structure matrix is constructed from the end-effector and the mechanism frame. Different cable quantities and arrangements yield several CDPM configurations. A configuration optimization approach for the CDPM is devised based on the installation space, end-effector geometry and load, motion DOFs, and the required range. This work analyzed the WCW and WFW of different CDPM configurations, incorporating posture variations to select the appropriate design. Moreover, the effects of the cable tension constraints and the distance between the attachment points and the end-effector vertices on the WFW are investigated. By balancing cost and performance, the approach selects appropriate cable tension constraints and attachment point locations on the end-effector.

In future work, we plan to extend the current methodology by incorporating cable dynamics and collision interference detection into the analysis, which are critical for ensuring the fidelity and safety of large-scale motion simulators under high-speed and heavy-load conditions. The dynamic behavior of cables, including elasticity and sagging, will be modeled to evaluate their impact on the end-effector’s trajectory tracking performance, rather than performing a full configuration check for each sample. Furthermore, collision detection algorithms will be developed to prevent interference between cables, as well as between cables and the end-effector or surrounding structures, during complex maneuvers. The above dynamic analysis will be integrated with desired simulator trajectories to assess the feasibility and accuracy of motion reproduction. Finally, experimental validation using a scaled or full-scale prototype will be conducted to verify the motion indicators and demonstrate the practical applicability of the proposed cable-driven parallel mechanism. Modeling cable elasticity and its effect on trajectory tracking accuracy will be addressed in our future work.

## Figures and Tables

**Table 1:** Geometric parameters of the cable-driven parallel simulator.

Geometric Parameters	Value
Length of Frame	20 m
Width of Frame	20 m
Height of Frame	11 m
Edge Length of Platform	2 m
Maximum Payload	1000 kg
Pitch Range	[−60°,60°]
Yaw Range	[−5°,5°]
Feasible Cable Tension	[1000 N, 28,000 N]

**Table 2:** The number of WCW points for different configurations.

Configuration	Number of WCW Points
(a)	5220
(b)	38,542
(c)	93,636
(d)	47,172
(e)	41,968
(f)	91,000

## Data Availability

Data are not publicly available due to proprietary manufacturing constraints but are available from the corresponding author upon reasonable request.

## Abbreviation

The following abbreviation is used in this manuscript:

CDPM	Cable-driven parallel mechanism
